# A Limited Role for Suppression in the Central Field of Individuals with Strabismic Amblyopia

**DOI:** 10.1371/journal.pone.0036611

**Published:** 2012-05-23

**Authors:** Brendan T. Barrett, Gurvinder K. Panesar, Andrew J. Scally, Ian E. Pacey

**Affiliations:** 1 Bradford School of Optometry & Vision Science, University of Bradford, Bradford, West Yorkshire, United Kingdom; 2 School of Health Studies, University of Bradford, Bradford, West Yorkshire, United Kingdom; The University of Sydney, United States of America

## Abstract

**Background:**

Although their eyes are pointing in different directions, people with long-standing strabismic amblyopia typically do not experience double-vision or indeed any visual symptoms arising from their condition. It is generally believed that the phenomenon of suppression plays a major role in dealing with the consequences of amblyopia and strabismus, by preventing images from the weaker/deviating eye from reaching conscious awareness. Suppression is thus a highly sophisticated coping mechanism. Although suppression has been studied for over 100 years the literature is equivocal in relation to the extent of the retina that is suppressed, though the method used to investigate suppression is crucial to the outcome. There is growing evidence that some measurement methods lead to artefactual claims that suppression exists when it does not.

**Methodology/Results:**

Here we present the results of an experiment conducted with a new method to examine the prevalence, depth and extent of suppression in ten individuals with strabismic amblyopia. Seven subjects (70%) showed no evidence whatsoever for suppression and in the three individuals who did (30%), the depth and extent of suppression was small.

**Conclusions:**

Suppression may play a much smaller role in dealing with the negative consequences of strabismic amblyopia than previously thought. Whereas recent claims of this nature have been made only in those with micro-strabismus our results show extremely limited evidence for suppression across the central visual field in strabismic amblyopes more generally. Instead of suppressing the image from the weaker/deviating eye, we suggest the visual system of individuals with strabismic amblyopia may act to maximise the possibilities for binocular co-operation. This is consistent with recent evidence from strabismic and amblyopic individuals that their binocular mechanisms are intact, and that, just as in visual normals, performance with two eyes is better than with the better eye alone in these individuals.

## Introduction

When visually normal humans view an object, the eyes move together so that the object of interest is simultaneously imaged on or near the fovea of both retinas because this tiny feature of the retina (diameter at foveal centre 0.2–0.3 mm, [1]) is responsible for acute vision. But bi-foveal imaging not only increases the level of detail that can be seen, it also provides the conditions that enable the highest levels of binocular co-operation to be achieved. Most notably, detailed information can be recovered about the relative distances of different objects from the observer owing to the fact that, due to their horizontal separation by around 6 cms, the right and left eyes receive very slightly different views of the world [2]–[4]. This depth-recovery feature is known as stereopsis and it is the aspect of human vision that is exploited by 3D-film makers to create the illusion of depth from imagery displayed on flat cinema screens.

**Figure 1:**
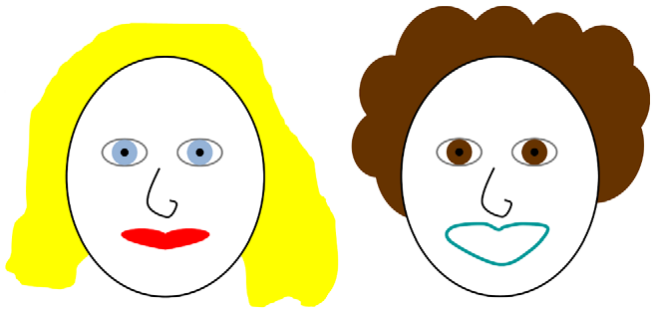
Illustration of the concepts of diplopia and ‘confusion’. Position yourself around 40 cm away from the figure. Place a pen in between your eyes and the figure. Hold it around 20 cm from your nose and stare at its tip. Because your eyes are over-converged for the faces, each face will appear doubled (diplopia). Visual confusion is produced by the superimposition of two faces, the right-hand version of the blue-eyed face and the left-hand version of the brown-eyed face. See text for further details.

In individuals with strabismus (a condition in which the eyes are misaligned eyes, prevalence ∼5% [5], [6]), only one eye is directed towards the object of interest. Individuals with a large manifest strabismus exhibit either no stereopsis or grossly-reduced stereopsis when tested clinically [6]. Aside from the effects on stereopsis, strabismus presents two additional, and potentially more serious, difficulties. These are termed diplopia and confusion (Figure 1) [7]. Firstly, since the object of interest is imaged on a non-foveal area of the retina of the deviating eye, diplopia (commonly known as ‘double vision’) should arise because the same object of regard is perceived as having two spatial locations, one arising from each eye. Confusion arises from the fact that, because it is deviated, something other than the object of interest must be imaged on the fovea of the deviating eye. The images formed on the fovea of the deviating and non-deviating eyes are dissimilar and thus cannot be fused (Figure 1). Strabismus frequently co-exists with amblyopia (popularly known as ‘lazy eye’), a condition in which there is reduced vision despite optimal optical correction and the absence of pathology of the eye or visual system [8], [9]. Indeed between one-half and two-thirds of amblyopes have strabismus [8], [10]. Like strabismus, amblyopia also presents the visual system with a binocular problem because confusion could arise from the superimposition of a blurred or distorted image from the weaker eye with a clear image from the fellow eye. This study is concerned with the extent to which objects presented to the deviating eye of those with both strabismus and amblyopia (a condition called ‘strabismic amblyopia’) reach consciousness awareness.

Fortunately for those affected, the visual system has at its disposal some defence mechanisms to avoid the problems of confusion and diplopia. One of the proposed mechanisms is known as suppression, the effect of which is to render invisible objects from all or part of the visual field of the deviated eye. Suppression represents a ‘switching off’ of vision from one eye that arises in binocular viewing conditions only; as soon as viewing becomes monocular (e.g. when the fellow eye is closed), suppression disappears and objects that were previously invisible suddenly appear. Suppression is thus conceptualized as a mechanism for suspending visual perception [11] thus preventing information from the deviating/weaker eye from reaching conscious awareness [6], [7], [12].

Starting with von Graefe in 1854 [13], a long series of studies have examined the size and position of retinal locations that are suppressed in strabismic individuals and the depth of that suppression (see [14], [15] for historical reviews). While clinical dogma is that diplopia and confusion are eliminated by suppression, examination of the literature reveals a mixed pattern of results in which there is suppression of retinal areas corresponding to either the diplopia-point or the confusion-point, or larger retinal areas that encompass both the diplopia- and confusion- points (Figure 1) [14]–[16]. Possible reasons for such differing results are discussed below.

This study is concerned with the fate of objects imaged across the central visual field of strabismic amblyopes, not just in the small areas corresponding to the diplopia- and confusion-points. Although fewer studies have mapped the extent and depth of any suppression across the central field, the results are no less variable than those which emerged from studies of more localised retinal areas (for review see [16]). For example, Pratt-Johnson and colleagues [17], [18] reported that strabismic suppression generally involves the whole of the visual field of the deviating eye except for its monocular temporal crescent. Jampolsky [19] found that suppression was present in one half of the retina only and Sireteanu & Fronius [20] described suppression zones that were surrounded by areas where binocular co-operation was present. Joosse et al. [21] found suppression zones in only 5 of their 14 subjects with micro-strabismus. While the presence and extent of suppression in strabismic subjects appears to depend upon both the size and direction (esotropia versus exotropia) of the deviation [14], [15], [21], [22] and on whether or not amblyopia is also present [12], individuals with almost identical clinical presentations can show hugely different depths and/or extents of suppression [15], [21].

This begs the question of whether or not suppression is a core characteristic of vision in individuals with strabismus, amblyopia or both (i.e. strabismic amblyopia). If suppression is not a pervasive feature of vision in individuals with these conditions, how then are the negative visual consequences (discussed above) avoided? We return to this question in the discussion.

What could account for the huge variety of findings in the literature concerning the presence/absence of suppression in strabismic individuals? The main reason appears to be that suppression is extremely challenging to reveal and measure, and the method of measurement can profoundly influence the result obtained. The enigma of suppression stems from the fact that, in order to reveal its presence, objects need to be separately presented to the right or left eye under conditions where both eyes are open. A number of different experimental approaches have been developed to facilitate this requirement, including coloured or polarising filters, mirrors and haploscopic presentation devices (see [14]–[16] for reviews).

The impact of measurement method on the result was elegantly demonstrated by Mehdorn [23] who could find no evidence for suppression in eight individuals using stereoperimetry, a technique which establishes if stereopsis can be revealed when disparate images are presented separately to the two eyes. The idea is that the perception of depth can only arise if the right and left eye percepts are combined; hence the depth percept cannot arise if suppression exists at that visual field location and presenting targets either in front or behind the reference plane provides a psychophysical means of testing that depth is in fact being seen.

While Mehdorn's [23] subjects showed little or no suppression using stereoperimetry the same individuals did exhibit clear central suppression when tested using more conventional, dissociating perimetric techniques. Since suppression acts to eliminate binocular co-operation, the fact that stereopsis was demonstrated in regions which other techniques indicated were suppressed was taken to mean that these regions of ‘suppression’ were nothing more than an artefacts of testing. This idea is not entirely new because and several previous studies had highlighted the critical nature of the test method to the outcome of suppression testing [14]–[16], [25].

All of Mehdorn's study participants had a particular form of strabismus, namely microstrabismus. This is a clinically discrete form of strabismus which is diagnosed when the angle of misalignment is small, there is no movement of either eye when its fellow is covered, and when an area of the retina other than the fovea is used for fixation (‘eccentric fixation’) [26]. Mehdorn's results in microstrabismics were replicated in a recent study that again employed stereoperimetry [24].

The technique of stereoperimetry for the assessment of suppression is strabismics can only be employed in those who possess some residual stereopsis. For this reason many individuals with strabismus [27], amblyopia [28], or strabismic amblyopia will not be suitable for testing using stereoperimetry. Here we describe a novel method for assessing the depth and extent of suppression in individuals with strabismic amblyopia. Our method features minimal dissociation and thus conditions which very closely resemble habitual viewing. As with stereoperimetry results in microstrabismics [23], [24], our results indicate a distinct lack of suppression in strabismic amblyopes more generally. On this basis, we suggest that the deviating eye in individuals with strabismic amblyopia (i.e. not only those with micro-strabismus, [15], [23], [24], [29]) may make a greater contribution in habitual viewing than previously thought.

## Results

### Effect of Amblyopia

**Figure 2:**
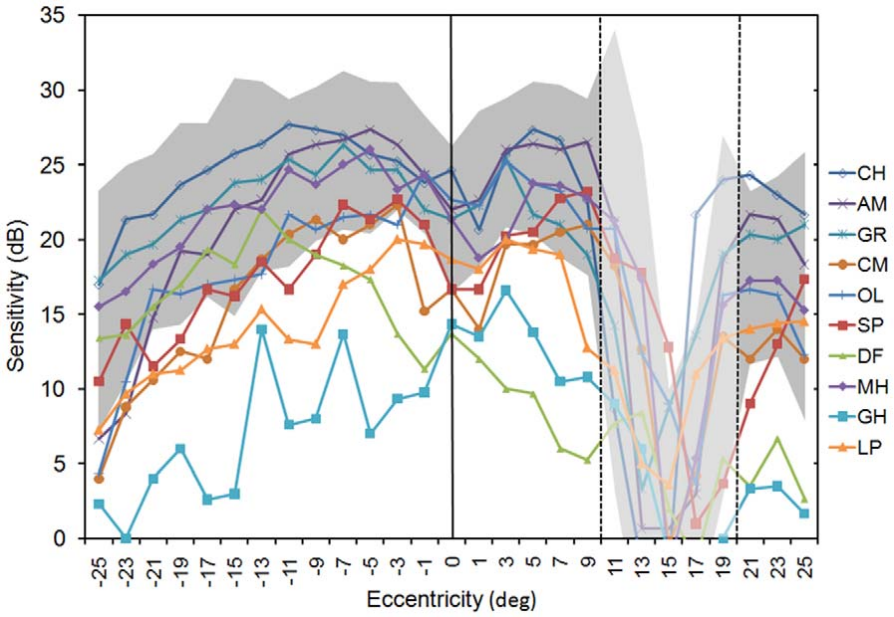
Data from individual amblyopic eyes compared with the range of sensitivities found in the controls' non-dominant eyes (grey region). ‘Sensitivity’ (y-axis) refers to the ability to detect the blue stimulus against the yellow background. Sensitivity is measured in decibels (dB) and, consistent with standard, white-on-white perimetry, a higher sensitivity indicates better performance. Error bars represent ±1 standard deviation of the mean. All left eyes were mirrored horizontally to allow comparison with the right eyes across the groups. The pale area between the vertical dotted lines (eccentricities +10 to +20 deg) corresponds to the region of the visual fields affected by the physiological blind spot for each participant.

All of our participants had strabismic amblyopia (Table 1). Since amblyopia is present under both monocular and binocular viewing conditions, we first examine whether the performance of our strabismic amblyopes was reduced by their amblyopia by comparing the monocular sensitivity of eyes with strabismic amblyopia with the monocular sensitivity of visual normals. This comparison is shown in Figure 2 where it can be seen that the sensitivity of the majority of eyes with strabismic amblyopia did not differ relative to the sensitivity of the non-dominant eye of visual normals (Table 1). This is consistent with previous studies [30], [31] showing that amblyopia does not result in a dramatic loss of sensitivity on perimetric tasks, although previous studies have used white-on-white targets, not blue-on-yellow ones as we used here. Three of our strabismic amblyopes (Figure 2: participants DF, GH, & LP) do show a clear of loss of sensitivity under monocular conditions relative to visual normals but these were amongst our oldest participants. Yellowing of the crystalline lens with age will account for at least part of their sensitivity loss due to greater absorption of short wavelength light [32]. This explanation is supported by the fact that monocular sensitivities for these subjects were also reduced when they viewed with their dominant eye (not shown).

**Table 1:** Clinical details of strabismic amblyopic participants.

Initials	Age	Hab RVA	Hab LVA	Dom eye	Optimal RE Rx	Optimal LE Rx	Optimal VA of AE	Optimal VA of FE	Near angle	Bagolini	EF	Stereoacuity	Habitual Rx
AM	21	0.30	−0.10	L	+1.00/−0.75×15	+1.00 DS	0.30	−0.10	6Δ RSOT	R Sup	1°N	300″	Optimal
CH	20	−0.10	0.48	R	Plano	+4.50 DS	0.40	−0.10	6Δ LXOT	L Sup	1.5°T	600″	None
CM	26	−0.02	0.50	R	+5.75/−1.75×110	+6.00/−2.00×68	0.50	−0.02	10Δ LSOT	L Sup	2.5°N	Negative	Optimal
DF	41	−0.20	0.20	R	+2.00 DS	+3.75/−0.25×65	0.18	−0.20	4Δ LSOT	No sup	0.5°N	600″	RE: +2.00 DS, LE: +2.00 DS
GH	54	−0.10	0.58	R	+1.25/−0.25×90	+4.75/−0.25×80	0.54	−0.10	14Δ LSOT	L Sup	1.5°N	Negative	RE: +0.50 DS, LE: +4.00 DS
GR	23	0.20	−0.10	L	+6.00/−3.25×85	+4.00/−2.00×75	0.20	−0.10	4Δ RXOT	No sup	0.5°T	300″	Optimal
LP	56	0.80	0.18	L	+2.00 DS	+3.50/−3.00×45	0.80	0.18	10Δ RXOT	R Sup	2°T	Negative	Optimal
MH	46	0.30	0.00	L	+3.00 DS	+2.75 DS	0.30	0.00	10Δ RSOT	R Sup	2°N	Negative	Optimal
OL	28	0.84	0.00	L	+4.25/−3.50×170	+1.50/−1.25×175	0.80	0.00	45Δ RXOT	R Sup	2.5°T	Negative	RE: +2.75/−3.50×170, LE: 0.00/−1.25×175
SP	36	0.42	0.00	L	+1.75/−0.50×5	−0.50/−0.25×10	0.38	−0.06	10Δ RSOT	R Sup	2°T	Negative	Optimal

Monocular visual acuity (VA, logMAR) are presented for left (L) and right (R) eyes under both habitual and optimal refractive conditions for the amblyopic eye (AE) and fellow eye (FE). The refractive correction worn habitually by each participant is also shown as Optimal (same as optimal refaction), None (no correction worn) or detailed in other cases. Oculomotor status and measurements at near viewing are presented and stereoacuity is given in seconds of arc (negative indicates worse than 600″) on the Frisby stereo acuity test. Eccentric fixation (EF) was measured in strabismic amblyopes using an interocular afterimage transfer method and the results are presented in degrees nasal (N) or degrees temporal (T). In visual normals, ocular dominance was determined with the Kay-pictures Dominant Eye Test (www.kaypictures.co.uk/dominant.html) and was classified as the eye that was used for sighting on at least two of the three presentations. The presence of abnormal retinal correspondence (ARC) was inferred from the results of the Bagolini lens test. On this basis only two participants (DF & GR) were diagnosed as displaying ARC. All four extropes are primary rather than consecutive exotropes. Abbreviations: AE = Amblyopic Eye, FE = Fellow Eye, Dom = Dominant Eye, XOT = Exotropia, SOT = Esotropia, Sup = suppression, DS = Dioptre Sphere, Δ = Prism Dioptres.

### Results in Visual Normals

To establish the depth and extent of any suppression, the sensitivity of an eye is measured when its fellow is open with minimal dissociation and this is compared to the sensitivity when the fellow eye is occluded. Suppression is present if performance of the eye is reduced when its fellow is open and viewing relative to when it is occluded.

**Figure 3:**
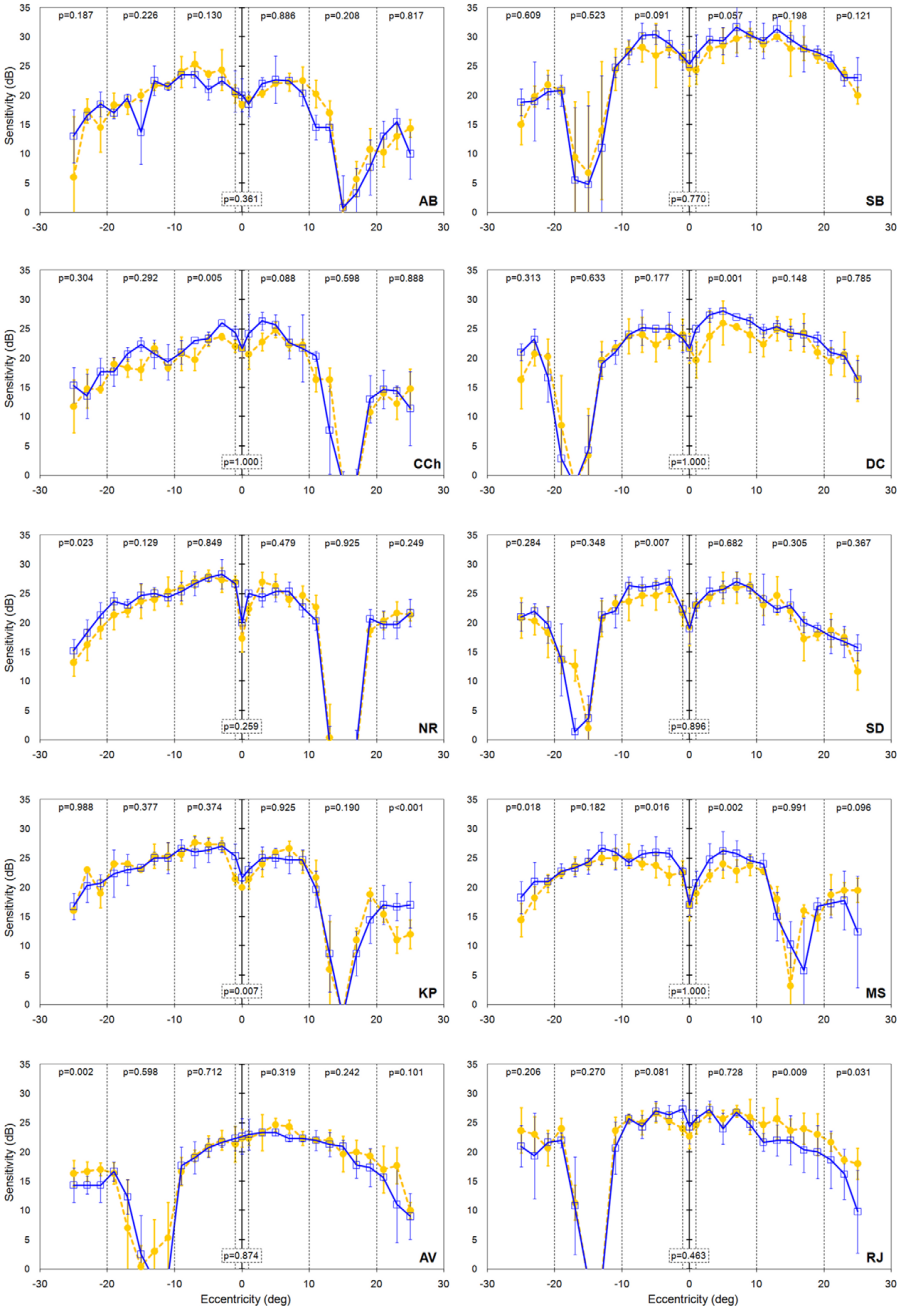
Sensitivity of the non-dominant eye of the 10 visually normal controls when the dominant eye viewed through the yellow filter (orange filled circles, dashed line) or had translucent occlusion (blue open squares, solid line). Error bars represent ±1 standard deviation of the mean. Negative values on the eccentricity axis correspond to visual field locations to the left of the fixation point. P-values are from the regression analysis within each region separated by the vertical dotted lines.

Before examining the results from our strabismic amblyopes the results from our visually normal participants are first considered. Figure 3 shows results for all ten controls. For each visual normal, sensitivity of the non-dominant eye was established when the dominant eye (Table 1) was occluded (blue curve) and when the dominant eye was open as normal but unable to detect the blue stimulus due to the presence of the yellow filter (orange curve). Figure 3 also shows the results from regression analysis of a region-by-region comparison of the sensitivities of the non-dominant eye under the two different viewing conditions. Two of the visual normals showed sensitivities that did not differ under the two conditions of viewing at any of the retinal locations (Figure 3: participants AB & SB). The remainder of the visual normals showed at least one region where sensitivity of the non-dominant eye was found to depend on the method used to prevent the non-dominant eye from detecting the stimulus. In six of the normals, there was evidence of suppression in one (n = 4; Figure 3: participants CCH, DC, NR & SD), two (n = 1, Figure 3: participant KP) or three (n = 1, Figure 3: participant MS) of the regions. In each case, the amount of suppression, although statistically significant, was small (<5 dB). In the remaining two visual normals, sensitivity was better when the dominant eye was open and viewing compared to when it was occluded in one (Figure 3: participant AV) or two (Figure 3: participant RJ) retinal regions. However, these differences were again small. In visual normals, rivalry between the eyes represents the most likely explanation why non-dominant eye performance is reduced with the dominant closed relative to when it is open.

### Presence of Suppression in Strabismic Amblyopia?

**Figure 4:**
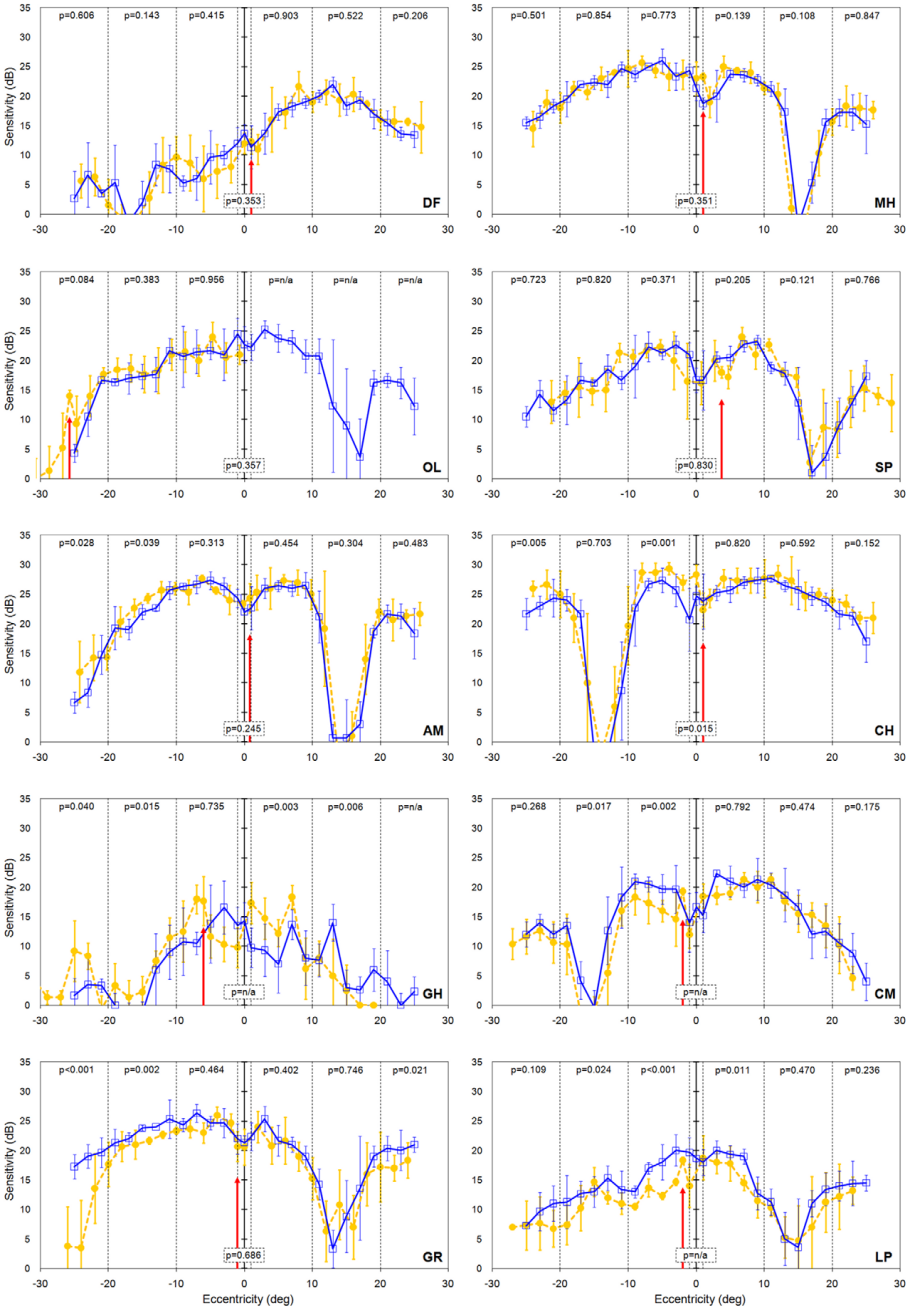
Sensitivity of the amblyopic eye of the 10 strabismic amlyopes when the dominant eye viewed through the yellow filter (orange filled circles, dashed line) or had translucent occlusion (blue open squares, solid line). Error bars represent ±1 standard deviation of the mean. Negative values on the eccentricity axis correspond to visual field locations to the left of the fixation point. P-values are from the regression analysis comparing the two viewing conditions within each region separated by the vertical dotted lines. P = n/a signifies that no data are present from the yellow filter viewing condition due to the horizontal adjustment described in the text. The vertical red arrow indicates the location which corresponds to the straight ahead position of the strabismic eye when the dominant eye was fixating through the yellow filter.

The results from our visual normals provide a useful backdrop against which to consider results from our strabismic amblyopes which are presented in Figure 4. In four strabismic amblyopes (Figure 4: participants DF, MH, OL & SP) the sensitivity of the deviating eye was unaffected by the occlusion status of the fellow eye; in other words, there was no evidence of suppression. In three strabismic amblyopes (Figure 4: participants AM, CH & GH), sensitivity was *better* in two or three regions by a small amount (typically ∼5 dB) when the fellow eye was open and viewing compared to when it was occluded. Again, this is not suppression because the suppression would cause the sensitivity to be reduced when the fellow eye is open and the dissociation between the eyes is minimal.

**Figure 5:**
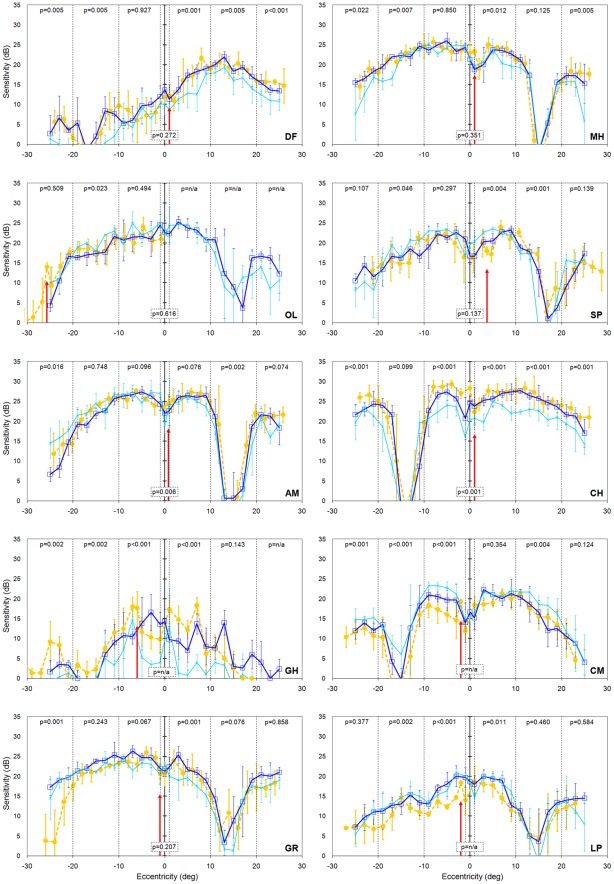
Sensitivity of the amblyopic eye of the 10 strabismic amblyopes when the dominant eye viewed through the yellow filter (orange filled circles, dashed line), had translucent occlusion (dark blue open squares, solid line), or had total occlusion (light blue crosses, solid line). Error bars represent ±1 standard deviation of the mean. Negative values on the eccentricity axis correspond to visual field locations to the left of the fixation point. P-values are from the regression analysis comparing the yellow filter and total occlusion viewing conditions within each region separated by the vertical dotted lines. P = n/a signifies that no data are present from the yellow filter viewing condition due to the horizontal adjustment described in the text. The vertical red arrow indicates the location which corresponds to the straight ahead position of the strabismic eye when the dominant eye was fixating through the yellow filter.

Only three of the ten strabismic amblyopes show any evidence for suppression. Subjects GR and LP show suppression in three regions and subject CM shows suppression in two regions (Figure 4). The literature would suggest the region most prone to suppression is the area between the straight ahead position and the fovea of the strabismic eye [6], [14]–[16]. This corresponds to the region between 0 degrees eccentricity and the vertical red arrow in Figure 4 and is observed for participants CM and LP but not GR. The magnitude of the suppression in our three cases is small, typically of the order of 5 dB, and broadly similar to the amounts of suppression reported by Joosse *et al* [21] in individuals with micro-strabismus. In order to ensure that the failure to reveal large or deep areas of suppression in our strabismic amblyopes was not due to the means we used to occlude the fellow eye of our strabismic amblyopes, we also gathered data sets for the condition when the fellow eye was taped shut. These results are presented in Figure 5 for all strabismic amblyopes. Irrespective of the method used to occlude the fellow eye (total occlusion or translucent occluder), suppression arose infrequently and to a small extent. For the three subjects who originally showed suppression, the magnitude of suppression became larger in subject CM, but it was unchanged in subject LP and suppression was present in fewer retinal regions in subject GR (Figure 5). For the other seven subjects who originally showed no signs of suppression, the results obtained when the fellow eye was taped shut were often not identical to those obtained when a translucent occluder was used (e.g. Figure 5: participants CH, GH & DF) but again there was an absence of suppression.

### No Dependence on Type of Strabismus or Depth of Amblyopia

Whereas others [15], [21], [22] have reported differences between suppression in exotropes versus esotropes, no such pattern is present in our results; of the three subjects who show suppression, two are exotropes (LP and GR, Figure 4) and one is an esotrope (CM, Figure 4). However, our participant numbers with suppression are too small to make a meaningful comparison here. Similarly we did not find a relationship between amblyopia depth and presence or depth of suppression. Of the three amblyopes who showed suppression, one subject (GR) had mild amblyopia and two had moderate to deep amblyopia (LP and CM) (Table 1).

## Discussion

Our principal finding is that the deviating/weaker eye of strabismic amblyopes was not suppressed at all in 70% of our strabismic amblyopes. In the remaining 30% who did show suppression, the regional extent of the suppression was small, and the associated reduction is sensitivity was small (<5 db). This serves to emphasise our main point that suppression may not play a prominent role in the vision of strabismic amblyopes. This finding runs contrary to the widely-held view that suppression is a core mechanism that prevents confusion and diplopia (Figure 1) in individuals with strabismus and strabismic amblyopia (e.g. [6], [17]–[19]).

Interestingly, six of the ten visual normals exhibited suppression (i.e. a *greater* proportion than in our strabismic amblyopes), though again, when it arose it was narrow in extent and shallow in depth. At first glance it is strange to find any suppression in visual normals. However, it is not uncommon to find limited ‘suppression’ in normals (e.g. [33], [34]) but its existence is likely to be related more to eye dominance effects and/or binocular rivalry during testing than genuine inter-ocular suppression. Our conclusion again is *not* that there is more suppression in normals than in strabismic amblyopes but that we find we extremely limited evidence for suppression in our strabismic amblyopes, even less than we found in our controls.

When suppression is investigated, the method of measurement is crucial to the outcome (see Introduction). Might our method of measurement have lessened the chances that we would find suppression? Techniques featuring greater levels of dissociation are less likely to reveal suppression because they differ to a greater extent from habitual viewing conditions [15], [23], [24], [35]. Compared to most other methods (reviewed in [14]–[16]), our experimental conditions featured minimal dissociation. In fact our experiment provided the right and left eyes with virtually identical imagery and these are precisely the conditions which should reveal suppression if it is present. The fact that we find so little evidence for suppression in our task calls into question the extent to which individuals with strabismic amblyopia rely upon suppression to avoid confusion and diplopia.

Our results are consistent with results of studies by Campos [15], Mehdorn [23] and Otto et al [24]. The latter studies used the technique of stereoperimetry in subjects with micro-strabismus and both concluded that the fixation-point suppression ‘scotomas’ reflect a perimetric artefact. Von Noorden [6] and others [15], [25] have also raised concerns about the validity and consistency of suppression results in strabismic individuals. Mehdorn [23] claimed that stereoperimetry was uniquely suited to investigate the presence or absence of suppression in strabismics (but only those with some degree of residual stereopsis) because objects that are presented only to the deviating eye (i.e. as in normal perimetric approaches to studying strabismic suppression) will be suppressed since the background information received by the non-deviating eye will always override the stimulus presented to the deviating eye. According to Mehdorn [23] therefore, suppression is an inevitable finding unless the same stimulus is presented to both eyes. Campos' [15] view is that only targets that can be fused and presented in non-artificial conditions are suitable for investigating suppression. Our findings differ from Mehdorn [23] and Campos [15] because, although like them we find very little evidence for suppression, our results were obtained in strabismic amblyopes the majority of whom did not have clinically measurable stereopsis and under conditions in which the stimulus to be detected was presented only to the deviating eye.

Our results are also similar to those of Joosse et al. [21] who, like us, employed a binocular perimetric approach and who also found suppression in only around one-third (5 of 14) of their microstrabismic participants, of whom all were of the convergent type. However, our results differ from a study by the same group [22] who, using the same methodology, found large-areas of suppression in 75% (12 of 15) of their participants with divergent strabismus. As indicated earlier, previous studies [15], [21], [22] have noted differences in suppression between convergent and divergent cases of strabismus but we did not find such a difference. However, our participant numbers are too small to allow us to make a definitive statement about differences between convergent (n = 6) versus divergent strabismus (n = 4, Table 1).

Might other aspects of our stimulus presentation have masked a genuine presence of suppression? The task was to detect a blue stimulus on a yellow background. Might this have influenced our findings? We do not believe so for two reasons. Firstly there is no evidence that colour vision or the processing of coloured stimuli is different relative to achromatic/luminance information in individuals with strabismus or amblyopia [36]–[38] and Figure 2 shows that sensitivity on this task for the majority of our study participants was within normal limits. Secondly, and more importantly, suppression is diagnosed from a comparison of sensitivity of the deviating eye with the fellow eye occluded compared to when the fellow eye is open. Hence, the absolute performance level on the task is less important to the diagnosis than a comparison of sensitivities of the weaker eye under open- versus closed-conditions of the fellow eye.

It is true to say, however, that certain aspects of our stimulus presentation may have made the finding of suppression less likely. For example, the temporal profile of the stimulus onset and offset had a square-shape and this may yield less suppression compared to temporal profiles where the onset/offset is more gradual [39]. Another aspect relates to the short (200 msec) presentation duration employed by us. De Bulsunce and Sireteanu [40] examined the time course of suppression in individuals with amblyopia some of who also had strabismus. While considerable inter-subject differences existed, a general finding was that at long presentation durations (e.g. 1000 msec and above), all of their subjects suppressed the pattern presented to their weaker/deviating eye. We did not vary the presentation duration here because this presents difficulties in a simple yes/no detection task. Furthermore when stimuli are presented eccentrically, stable fixation is crucial and short presentation durations are needed to avoid saccadic eye movements to directly view the eccentrically-presented stimulus. However, we acknowledge that it is certainly possible that we would have found more suppression (more cases, more extensive and/or deeper) had we presented our stimulus for a longer duration. Having said this, Otto et al. [24], whose basic stimulus presentation duration was 100 msec in their task, found no deterioration in stereo-resolution when the duration of presentation was increased to 2000 msec and this corresponds with what was previously reported by Mehdorn [23] who used even longer presentation durations (up to 4 seconds). Another possible reason for our failure to reveal suppression in our strabismic amblyopes relates to the fact that the stimulus to be detected was presented against a uniform background. This absence of form may have masked suppression that might exist when more complex and variable imagery appeared at different visual field locations, as in the case of habitual viewing. A number of studies have examined how the pattern of strabismic suppression depends upon the nature of the stimulus presented to the non-deviating eye [35], [34], [41]. As is so often the case in the suppression literature, the results are somewhat equivocal. Some studies [33], [41] report that the sensitivity of the deviating eye is affected by the stimulus presented to the non-deviating eye. However, Freeman & Jolly [34] found the complete opposite. Unlike in visual normals, they found that the results they obtained in their strabismic subjects were independent of the stimulus presented to the fellow eye. Based upon our findings and an examination of existing literature we cannot rule out the possibility that the uniform field that we employed was responsible for the fact that we found so little evidence for suppression in our strabismic amblyopes. However we do not believe the uniform field explanation because (i) participants did not view a structureless field. Rather, when deviating eye's sensitivity was being assessed the fellow eye viewed the Humphrey Field Analyzer's diamond fixation target (angular subtense of 2.86 degrees); (ii) As a result of (i) the deviating eye was in its habitual, anomalous motor position and for this reason, if suppression normally exists, then one might expect it to engage when the deviating eye is in its deviated state; (iii) as indicated above there is evidence that suppression effects in strabismics are not governed by the pattern that is viewed by the fixing eye [34] and recently [42] in intermittent exotropes it has been shown that retinal images simulating the exodeviation reliably trigger neuronal mechanisms that prevented diplopia, even when the eyes were correctly aligned. Together this evidence suggests that if more suppression existed in our strabismic amblyopes we should have found it. However, while we believe that our basic finding of limited suppression is not dependent on our method of measurement, we acknowledge that a comparison of our approach with other techniques (in particular those which test binocular co-operation more directly, e.g. motion integration [28] or stereoperimetry [23], [24]) in the same individuals is now warranted.

Our failure to find compelling evidence for suppression in this task contrasts with the results of clinical suppression testing that we carried out on all our participants (Table 1). We used Bagolini lenses to test for suppression [6]. This test features two lenses, one placed in front of each eye. The lenses contain very fine striations which cause a spot of light to appear elongated. The lenses are oriented so that the elongation is orthogonal in the two eyes, meaning that individuals without suppression see the spot of light with two complete, crossed lines. Our Bagolini test results are shown in Table 1 and they indicate that eight of our ten strabismic amblyopes showed suppression on this test, contrary to what we found in our blue-on-yellow detection task (3 of 10). Why do our clinical and laboratory-based tests of suppression yield such different results? We do not know the answer to this question with certainty but the fact that the Bagolini lens test presents the stimuli for a long duration and that it is a test of suppression at the fixation point are two likely explanations. As indicated, our blue-on-yellow stimulus presentation was extremely short and we tested for suppression across the central 50 degrees of visual space. Interestingly, both Mehdorn [23] and Otto et al [24] also noted that their subjects who showed no suppression on stereoperimetry testing exhibited fixation-point suppression on the Bagolini test. Differences between the results of standard, clinical versus laboratory-based tests of suppression found here and in previous studies (e.g. [24]) emphasize the importance of the method used to test for suppression and the need for more detailed characterisation of the stimuli and test conditions that do and do not give rise to suppression in those with strabismus, amblyopia or both.

Our view is not that there is a complete absence of suppression in individuals with strabismic amblyopia, merely that suppression is not ubiquitous nor inevitable in these individuals. If this is correct, it follows that their binocular co-operation may be greater than imagined. While suppression of the deviating/weaker eye represents one mechanism that the visual system has at its disposal for avoiding symptoms arising from strabismus, it is not the only mechanism available (see below).

All of our participants had both strabismus and amblyopia. These conditions are not thought to be independent with strabismus generally considered to be a cause of amblyopia [6], [43]. The mechanism via which strabismus leads to amblyopia is thought to involve long-term chronic suppression of the deviating eye [6], [20], [44]. Examining the prevalence and depth of any suppression in individuals with strabismic amblyopia may thus shed light on the nature of the link between strabismus and amblyopia [45]. For example, if suppression is not a pervasive feature in strabismic amblyopes this could signal that suppression to avoid the consequences of strabismus did not play a part in generating the amblyopia. As with the suppression literature in general, the literature on the link between amblyopia/strabismus and suppression is equivocal. Holopigian et al. [12] found little suppression in individuals with amblyopia but marked suppression in strabismics without amblyopia. They reasoned that when an eye is amblyopic there is no longer a need for strong suppression of that eye by the contralateral eye. Freeman et al [46] also found a limited contribution of suppression to the visual acuity in strabismic amblyopes. Others however find that amblyopia is not only associated with suppression, but the depth of amblyopia correlates positively with the degree of suppression [20], [33], [44], [47], [48]. These findings are usually interpreted as evidence that the suppression was instrumental in generating the amblyopia. No such inference can be drawn from our results because we find so little suppression in our strabismic amblyopes.

If there is no suppression in strabismic amblyopia, why then do these individuals not report diplopia and/or confusion? (Figure 1). While suppression of the deviating eye represents one mechanism that the visual system has at its disposal for avoiding symptoms arising from strabismus, it is not the only mechanism available. Instead of suppression, the visual system may re-map the usual relationship between the two eyes with the aim of enabling the eyes to functionally cooperate despite their misalignment. This re-mapping is termed anomalous retinal correspondence (ARC) [6], [13]. Suppression and ARC are therefore completely opposite because the former is designed to ‘switch off’ imagery that gives rise to troubling visual symptoms whereas the purpose of the latter is to make the most of an imperfect system. It is recognised that ARC and suppression can co-exist in the same individual. For example, Sireteanu and colleagues [49], [50] found evidence for suppression in central field of strabismics (including strabismic amblyopes) but ARC in more peripheral field locations. Our experimental test results, contrary to clinical (Bagolini) test results (Table 1), suggest a potentially greater role for ARC [51] and a lesser role for suppression in strabismic amblyopes. The reasons for the disconnect between clinical and experimental findings (discussed above) are as yet unresolved but the putative co-operation between the eyes that is suggested by the absence of suppression is supported by results showing that binocular mechanisms remain intact in individuals with strabismic amblyopia [52] and by studies which show motion stereopsis may exist for dynamically presented stimuli in those who exhibit no stereopsis on standard, static stereopsis testing [53]–[56].

Studies examining the presence/absence of suppression in strabismic amblyopes go hand in hand with studies examining their performance on everyday tasks. For example, if the deviating/weaker eye is largely or wholly suppressed then it would not be surprising to find that these individuals function like visual normals who close one eye. This is not what is found, however. In fact there is evidence that just, as in visual normals, two eyes are better than one in strabismic amblyopes [57]–[59] thus suggesting a useful contribution from the deviating/weaker eye.

In summary, suppression has generated research interest from a range of perspectives for over 150 years (e.g. [11], [13]–[16], [60]–[62]). Recent results strongly suggest that suppression does not play a prominent role in micro-strabismus [15], [23], [24]. Our results suggest that the same may be true in strabismic amblyopes more generally, thus calling into question the presence and role of suppression in strabismus and strabismic amblyopia.

## Methods

### Ethics statement

The tenets of Declaration of Helsinki were followed and the study had approval of the University of Bradford Ethics Committee, with written informed consent being obtained from all participants prior to their participation.

### Participants

A total of 10 adult strabismic amblyopes took part. Prior to participation all subjects underwent full eye examination and binocular vision assessment. Clinical details for each study participant are presented in Table 1. All of our participants exhibited a manifest strabismus on cover/uncover testing. We did not recruit participants who had microstrabismus with identity (i.e. a small angle strabismus that does not reveal itself on cover/uncover testing [63]). Ten visually normal control subjects also participated in the study.

### Protocol

Using the Humphrey Visual Field Analyzer (HFA, model 745i, Carl Zeiss Group), we assessed sensitivity to a narrow-band, blue light stimulus with a peak wavelength of 440 nm (Goldmann size III target, 0.43 degrees) presented on a 100 cd/m^2^ broadband (500–700 nm) yellow background with a stimulus duration of 200 msec. This blue-on-yellow detection task that we have employed was originally added to the HFA for the detection of earlier glaucomatous defects by comparison with white-on-white perimetry [64]. Our choice of this task was for different reasons, specifically because it provided a straight-forward means for assessment of the deviating eye's sensitivity in its habitual motor position.

This stimulus arrangement allowed the estimation of sensitivity under a variety of dissociative viewing conditions. The use of a yellow filter (010 medium yellow, Lee filters, www.leefilters.com/lighting/products/finder/act:colourdetails/colourRef:C4630710C3E644) over one eye provided a viewing condition in which each eye sees the same, apart from the blue light stimulus which is only seen by the eye without the filter; the absorption characteristics of the filter ensured that the blue stimulus was invisible to the eye with the filter. This form of dichoptic viewing was favoured as it allowed investigation of the thresholds from one eye with minimal effects on motor position; the filter permitted the subject to view inside the bowl of the HFA with both eyes open as normal with the deviated eye in its habitual, deviated position. Alternative dichoptic viewing arrangements with different methods of dissociation could potentially alter the habitual motor position of the eyes (e.g. mirror haploscopic methods) or introduce rivalrous conditions (e.g. red/green dissociation). The sensitivity of the deviating eye obtained with the yellow filter occlusion (i.e. with the fellow eye open) was compared to that with translucent occlusion (light perception only from the fellow eye) and full total occlusion (no input from the fellow eye) to give an indication of the effect of the fellow eye on the deviating/weaker eye's performance. Suppression was deemed to be present if sensitivity was poorer under habitual viewing (fellow eye viewing through the yellow filter) compared to conditions when the fellow eye is occluded.

Our use of a blue stimulus in this dichoptic viewing arrangement does not make the finding of suppression any less likely as colour and luminance discrimination have been shown to be similarly affected in amblyopia both psychophysically [34], [35] and using fMRI at the visual cortex [36] where suppression is assumed to occur.

The blue-on-yellow detection task that we employ is the same as in standard automated perimetry in which a higher sensitivity (measured in decibels (dB) indicates an ability to detect a dimmer blue stimulus. In order to map the suppression across the central visual field, sensitivity was assessed in the straight ahead position, and at two degree intervals from 1 to 25 degrees along a horizontal meridian on either side of the straight ahead position. In order to avoid the horizontal raphe, measurements were assessed two degrees above the horizontal midline and the small diamond fixation target was used throughout to aid fixation stability for conditions that involved fixation by the weaker eye. Each participant viewed the target through full aperture trial lenses, positioned in a lightweight trial frame, as determined by inputting their age and optimal distance refraction (Table 1) into the HFA software. Participants received standard perimetric instructions regarding the procedure to be followed using the HVFA. Specifically, they were asked to maintain fixation at the centre of the diamond target throughout and to press the button on the hand-held unit when a blue light was detected.

Fixation was monitored manually by the clinician using the video camera and by the eye-tracking device of the HVA and participants were reminded, as necessary, throughout the trial to fixate on the fixation target straight ahead.

The HFA full-threshold programme used estimates sensitivity at each stimulus location using a 4–2 dB staircase procedure: an initial crossing of threshold in 4 dB increments and a final crossing in 2 dB increments with the threshold designated as the last seen stimulus luminance. The short-term fluctuation option was enabled allowing the HFA to retest some stimulus locations either randomly or when the initial estimation of threshold deviated from that expected by comparison with neighbouring locations.

All participants undertook trial runs at the start of each data collection visit to ensure that the instructions were understood and that the participant was able to perform the task. The data from the trial runs were not included in the analysis. Data was collected for a total of six viewing conditions as part of a larger study. One run of each viewing condition was collected on three occasions separated by between two and seven days. Each run lasted approximately nine minutes during which sensitivity was estimated at each of the 27 stimulus locations. Combining the three runs for each viewing condition produced a mean sensitivity at each location from between 3 and 6 estimates (median 4). In order to counter fatigue effects across participants, the testing order for the viewing conditions was randomised for each participant but held constant over each visit. The data from three of the viewing conditions are presented here.

1. Yellow filter over the fellow eye. This prevented the fellow eye from seeing the blue stimulus thus allowing the sensitivity of the amblyopic eye to be determined with fellow eye open and able to detect all other form. In this viewing condition the fellow eye maintains the ability to see the fixation target and thus was responsible for the motor position of the two eyes. Data gathered with the filter was from the non-dominant eye in its habitual motor position.
2. Translucent occluder placed in front of the fellow eye. Although this abolished all form vision it still allowed light to enter the eye because the eye was open behind the occluder. This can be thought of as occlusion but reduces the interocular effect on thresholds that frequently occurs with the total occlusion described in 3 below [65], [66].
3. Full total occlusion of the fellow eye. The fellow eye was taped closed, and an opaque patch was placed over the eye. This occlusion method abolishes all form vision and virtually all light from entering the eye providing ‘pure’ monocular thresholds of the non-occluded eye but at the risk of being affected by Ganzfeld blankout.

The motor status of the weaker, habitually deviating eye is identical in conditions 2. and 3. because, irrespective of whether the dominant eye is occluded with a translucent or opaque occluder, the motor position of the non-dominant eye changed from its habitual position as to view the HFA's fixation target. What was the rationale for including two types of form occlusion of the fellow eye (2. and 3., above)? When the fellow eye of an individual with amblyopia (with or without strabismus) is covered, participants often report that viewing with the affected eye is difficult because it's as though the form-free, fellow eye percept is rivalling the amblyopic eye percept. In some amblyopes, this is more noticeable when a translucent occluder whereas for others, the phenomenon is more apparent when an opaque occluder is employed. Because the intention here was to compare sensitivity of the deviating eye when the fellow eye is open and unoccluded (yellow-filter) with the sensitivity when fellow eye is occluded, we wanted to ensure that we were not underestimating the depth, or even missing altogether the presence, of suppression due to the method of occlusion of the fellow eye.

A group of 10 non-amblyopic control subjects (age range 20–44, mean 30.7) also took part in the study and attended for three visits of data collection, from their non-dominant eye, with the yellow filter and translucent occlusion viewing conditions (1. & 2.) described above.

### Data Analysis

The data gathered in the three viewing conditions described above cannot be directly compared because of the different motor positions adopted by the weaker/deviating eye when the fellow eye views through the yellow filter (habitual position) compared to when the fellow eye was occluded with translucent or opaque occlusion. As a result, the data were analysed in the following way. Firstly, for each participant, the data from the yellow-filter condition were adjusted horizontally to align the sensitivity profiles using the reduction of sensitivity due to the physiological blind spot as a reference. The red arrow in Figures 4 & 5 marks the location which corresponds to the straight ahead position (i.e. the fixation point of the fellow eye) and thus indicates the degree of horizontal adjustment made in each case. In order to compare the different viewing conditions for each participant, data were then grouped into seven regions of the horizontal meridian tested:

−20 to −25 degrees from the straight ahead position
−10 to −19.9 degrees from the straight ahead position
−1 to −9.9 degrees from the straight ahead position
−0.9 to 0.9 degrees from the straight ahead position
- 1 to 9.9 degrees from the straight ahead position
- 10 to 19.9 degrees from the straight ahead position
- 20 to 25 degrees from the straight ahead position

A separate regression analysis (Stata version 9 1997, www.stata.com) was performed for each region, within each participant, to compare the translucent- and full-occlusion conditions with the adjusted yellow-filter condition. The model used all available individual sesnitivity values and the data from each condition was fitted with a second order polynomial function. Suppression was assumed to be present if the yellow-filter sensitivity was lower than the other condition with a criterion for statistical significance set at p<0.05.
